# Turbidimetric analysis on the Hitachi 705 using orosomucoid as a model

**DOI:** 10.1155/S1463924684000419

**Published:** 1984

**Authors:** E. F. Legg, M. Sullivan

**Affiliations:** Department of Clinical Chemistry East Birmingham Hospital Bordesley Green East Birmingham BP 5ST UK


Journal of Automatic Chemistry, Volume 6, Number 4 (October–December 1984), pages 206–209

### Turbidimetric analysis on the Hitachi 705 using orosomucoid as a model

**E. F. Legg* and M. Sullivan**

*Department of Clinical Chemistry, East Birmingham Hospital, Bordesley Green East, Birmingham B9 5ST, UK*

### Introduction

An Hitachi 705 programmable analyser was recently purchased for East Birmingham Hospital's clinical chemistry laboratory. Although the principal use of the instrument was to process traditional spectrophotometric assays, it was considered desirable that the new instrument should be sufficiently versatile to enable turbidimetric analysis to be performed.

The work-load for serum orosomucoid estimation was sufficiently high (40/week) to justify an automated approach to save both analytical time and consumable costs when compared to the, then current, use of the Mancini immunodiffusion technique.

The advantages of turbidimetric assay of proteins compared with alternative techniques has recently been reviewed [1]; unfortunately no published methods for turbidimetric analysis using the Hitachi 705 are available and it was unknown whether such techniques were possible using the Hitachi 705.

This work was initiated in order to discover if the Hitachi 705 could successfully perform turbidimetric analysis of orosomucoid.

### Materials

### *Reagents*

*Radial immunodiffusion plates*

Conventional radial immunodiffusion analysis of orosomucoid was carried out using gels impregnated with anti-orosomucoid antibody obtained from Hoechst UK Ltd, Salisbury Road, Hounslow, Middlesex, UK.

*Phosphate buffered saline*

Dissolve 3·58 g disodium phosphate dodecahydrate, 1·56 g sodium dihydrogen phosphate dihydrate, 1·8 g sodium chloride, 1 g sodium azide and 40 g of polyethylene glycol (PEG) 6000 in water. Adjust the pH to 6·8 and make up to 1 litre. The solution was filtered through a 0·22 $\mu$m millipore filter before use.

*Antibody solution*

Anti-orosomucoid antisera, purchased from DAKO, Mercia Brocades Ltd, Brocades House, Pyrford Road, West Byfleet, Weybridge, Surrey KT14 6RA, UK, was diluted in phosphate buffered saline and left for 30 min prior to filtration through a 0·22 $\mu$m millipore filter. Dilution of the antibody in the final solution was arbitrarily set at 1 in 30. The titre of the neat antisera was 300, i.e. 300 $\mu$g orosomucoid react with 1 ml antiserum to produce equivalence.

*Standardization*

The primary standard used was obtained from Hoechst and had an orosomucoid concentration in the range 0·9–1·1 g/l. A pooled, filtered and hepatitis-free serum was ascribed a value of orosomucoid concentration using radial immunodiffusion analysis by reference to the primary standard. This pooled serum then served as a secondary standard and was used throughout this study.

### *Apparatus*

A Pye Unicam 1800 spectrophotometer (Pye Unicam Ltd, York Street, Cambridge CB1 2PX, UK) was used for the derivation of optimized conditions of the orosomucoid assay.

The Hitachi 705 used in this study is a programmable discretionary analyser and has recently been described in detail [2 and 3].

All assays were carried out at 37°C.

### Methods

### *Manual procedure*

The technique adopted for studying the optimization of reaction conditions was a modification of the nephelometric method of Buffone and Lewis [4], but using a turbidimetric end-point mode of assay. Briefly, this consisted of preparing an initial 1 in 10 dilution of serum, control and standards in 9·0 g/l saline. The diluted sample was then added to both 2·5 ml phosphate buffered saline (blank) and 2·5 ml of diluted antibody (test). The absorbance difference at 340 nm between the test and blank solutions is proportional to the orosomucoid concentration which was calculated by reference to standards.

### *Semi-automated procedure using the Hitachi 705*

The manual procedure was further modified to enable its adaptation to the Hitachi 705. Unless otherwise stated, all sera were diluted 1 in 10 using a microprocessor-controlled syringe (BCL Dilutrend) by aspirating 50 $\mu$l of serum and dispensing it with 450 $\mu$l saline (9 g/l) into a 2 ml plastic vial. The prediluted sera were placed on the Hitachi sample carousel and the instrument set to aspirate 10 $\mu$l of prediluted sample, followed 40 s later by the addition of 350 $\mu$l of phosphate buffered saline (reagent 1). A blank absorbance reading was obtained just prior to the addition of 80 $\mu$l diluted antibody (reagent 2) and this was subtracted from a further absorbance reading obtained 4 min later.

* Author to whom correspondence should be addressed.

### Results

### *Optimization of conditions*

Using the manual technique described above, a series of antigen/antibody curves were obtained. Figure 1 demonstrates the effect of using different volumes (7, 10, 12, 15, and 20 $\mu$l) of a linearly related series of serum standards on absorbance. Conditions were selected so that the point of equivalence (peak of standard curve) occurred at a point well above the highest levels seen in pathological conditions (about 4·0 g/l). The adoption of this measure meant excess antibody was always present and that the well-known problems associated with antigen excess [1] were avoided.

The influence of the concentration of pH, PEG 6000, sodium chloride and buffer, on the amount of turbidity produced from a pooled serum sample were investigated—the results are shown in figure 2. As a result of this study, the following buffer conditions were selected:

(1) Sodium chloride concentration 1·8 g/l (31 mmol/l).

(2) PEG 6000 concentration 40 g/l.

(3) pH 6·8.

(4) Phosphate buffer concentration 10 mmol.

The speed with which maximum absorbance was achieved after addition of antibody for a serum orosomucoid concentration of 1·5 g/l is shown in figure 3. The general profile of the curve was similar at other serum orosomucoid concentrations varying from 0·5 g/l to 3·5 g/l. At 37°C, 10 min was the minimum time required for turbidity to reach a maximum under the test conditions.

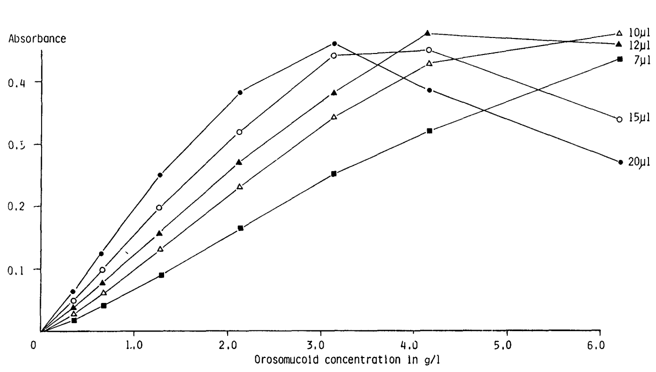

*Figure 1. Effect of increasing orosomucoid concentrations on absorbance using different serum volumes. All sera were prediluted 10 fold.*

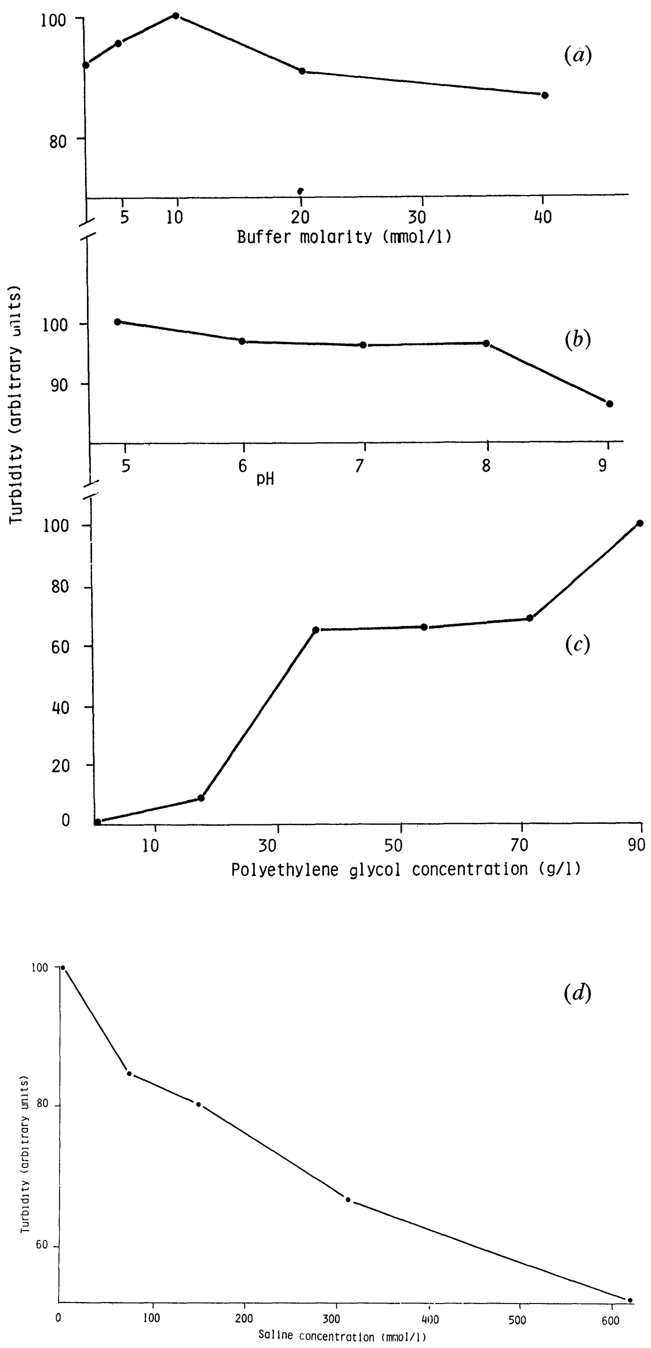

*Figure 2. Effect of buffer molarity (a), pH (b), polyethyleneglycol concentration (c) and saline(d) on turbidity.*

### *Modification of optimized conditions to the Hitachi 705*

Instrumental constraints prevented the optimized method being placed on to the Hitachi 705 in an unmodified form for two reasons.

Firstly, maximum turbidity did not occur at 37°C until 10 min had elapsed after addition of antibody (figure 3). Unfortunately, when using the Hitachi 705, it is not possible to delay the absorbance reading after antibody addition beyond 4 min.

The percentage of maximum turbidity reached at 4 min for different serum orosomucoid concentrations is shown in table 1. At all concentrations 86–91% of the maximum turbidity produced was obtained in 4 min.

Secondly, the Pye Unicam spectrophotometer produces absolute absorbances at 340 nm when using the optimized method, whereas the Hitachi uses bichromatic optics requiring measurement of absorbance differences by reference to a secondary wavelength. This facility cannot be disabled and renders the method less sensitive due to the lower absorbance obtained. A typical absorption spectrum is shown in figure 4. A secondary wavelength of 660 nm was chosen. These modifications (i.e. a 4 min incubation time and a secondary wavelength of 660 nm) were incorporated into the optimized method for analysis of orosomucoid on the Hitachi 705.

The conditions finally adopted for use on the Hitachi 705 are outlined in the Appendix.

*Table 1. Demonstrating percentage maximum turbidity reached at 4 min at different concentrations of serum orosomucoid.*

| Serum orosomucoid concentration g/l | % maximum turbidity at 4 min |
|---|---|
| 0·62 | 86 |
| 1·24 | 98 |
| 2·48 | 91 |
| 4·96 | 89 |

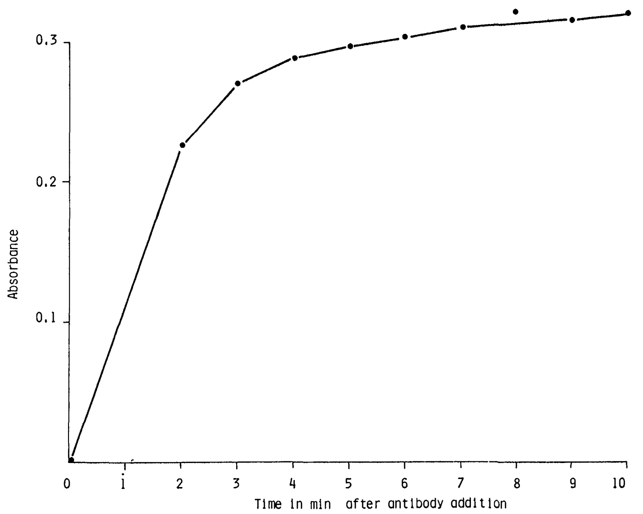

*Figure 3. Demonstrating development of absorbance (turbidity) with time.*

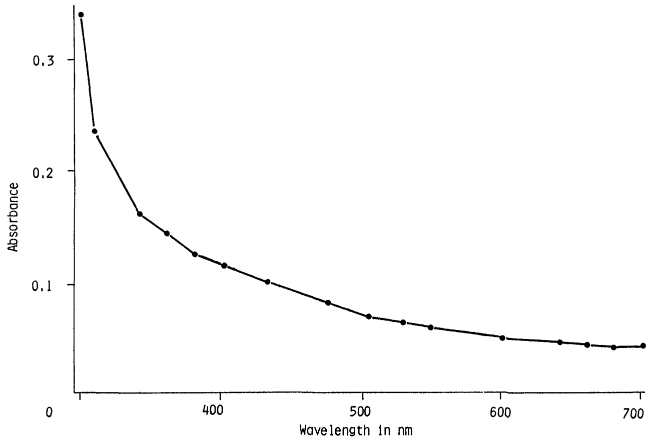

*Figure 4. Typical absorption spectrum of the turbidity produced under the proposed conditions of the test using the Pye Unicam SP1800. Concentration of orosomucoid in the serum was 1·2 g/l. Absorbances at 340 nm and 660 nm were 0·165 and 0·042 respectively.*

*Standardization*

A calibration curve is shown in figure 5. The graph is virtually a straight line, enabling single-point standardization to be carried out. If significant deviations from linearity occurred, it would be preferable to carry out multipoint standardization; unfortunately, this is not straightforward since the Hitachi 705 software will only accomodate single-point standardization.

There was, however, a less than 2% difference between the results obtained by (1) adopting single-point standardization and assuming linearity of the standard curve up to an orosomucoid concentration of 2 g/l; and (2) using multipoint standardization.

This problem can be circumvented by placing a series of standards in the test sample positions of the sample rotor and calibrating the Hitachi by single-point standardization. The values obtained for the serial standards in this way can then be plotted on the *y* co-ordinate against their known values in g/l, either manually or preferably via a microcomputer; the orosomucoid concentration of the test samples are then calculated from the standard curve in the usual way.

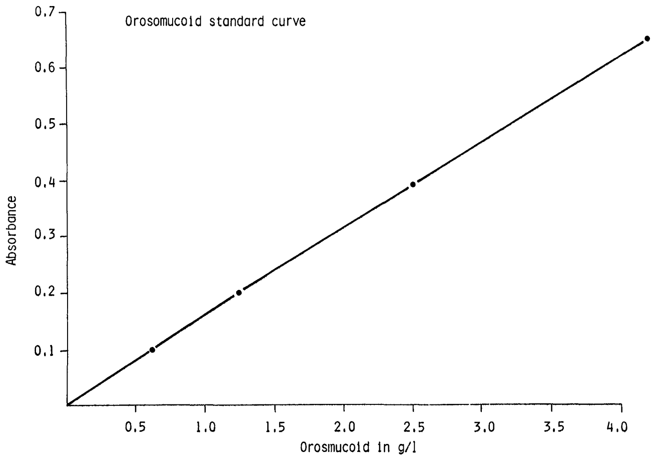

*Figure 5. Calibration curve for serum orosomucoid using the Pye Unicam SP1800 spectrophotometer.*

*Table 2. Comparison of turbidimetric and radial immunodiffusion methods of analysis for orosomucoid.*

| | Turbidimetry (Hitachi 705) | RID |
|---|---|---|
| Incubation/assay time | 15 min | 2 days |
| Pipetting | Mechanical | Manual |
| Reagent costs/test | 12·5p | 33p |
| Between-batch precision (CV) | 2·4 | 5·0 |
| Repeat analyses | Unnecessary | Required if orosomucoid >2·0 g/l |

*Comparison of Hitachi 705 method with radial immunodifussion (RID)*

A comparison of the two methods is shown in table 2. Radial immunodiffusion commonly costs between £0·80 and £1·00/assay when used according to the manufacturer's instructions, although many laboratories reduce the cost to £0·30–£0·40 by cutting extra wells in the antibody-impregnated gel; however, some pathological sera with markedly raised orosomucoid levels may overlap with other circles of antigen/antibody precipitation because of the increased density of the number of wells.

In contrast, turbidimetry, using the method described, costs £0·10–£0·15/assay and repeat analyses of pathological sera with elevated levels are unnecessary. In addition, results are available within 15 min, whereas the prolonged incubation times associated with radial immunodiffusion ensure results are unavailable for at least 24 h. The precision of the turbidimetric method is better than that of the Mancini radial immunodiffusion technique (table 2), while the correlation between the two methods is good (figure 6).

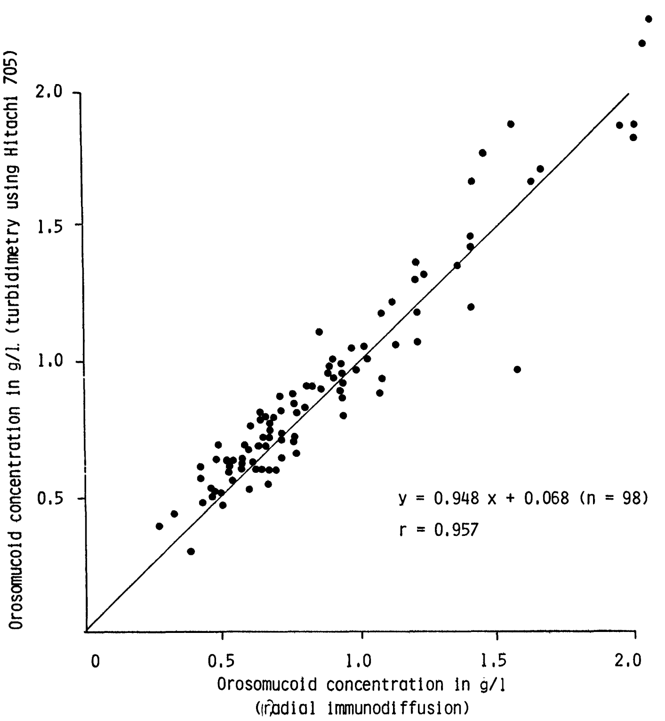

*Figure 6. Correlation of the turbidimetric and radial immunodiffusion (Mancini) methods of serum orosomucoid estimation.*

### Appendix

*Parameter settings for the estimation of orosomucoid on the Hitachi 705.*

| *Program 6* | *Chemistry parameters* |
|---|---|
| Test code | □ |
| Assay code | 1 (End point) |
| Sample volume ($\mu$l) | 10 (Serum prediluted 1 in 10) |
| R1*($\mu$l) | 350 |
| R2‡($\mu$l) | 80-□-YES |
| Wavelength 1 | 660 |
| Wavelength 2 | 340 |
| Reagent blank | † |
| Reagent blank concentration | 0 |
| Standard concentration | □-□-□ |
| Factor | † |
| Standard absorbance allowance | □ |
| Normal range L | 0·3 |
| Normal range H | 1·2 |
| Absorbance limit (rate) | 0 |
| Control ID No. | □-□-□ |
| *Program* 7 channel setting | |
| Channel No. | □ |
| Test code | □-□ |

□ Entered by operator.
† Computed by instrument.
*This solution contained 1·8 g/l sodium chloride, 40 g/l PEG 6000, 3·58 g disodium phosphate dodecahydrate, 1·56 g sodium dihydrogen phosphate dihydrate, 1 g sodium azide and had a pH of 6·8.
‡This solution is identical to reagent R1 but contains DACO anti-orosomucoid antisera (titre 300) at a dilution of 1 in 30.

### Discussion

Turbidimetric analysis is a well-known and simple technique used increasingly in clinical chemistry laboratories, which, in the end-point mode, can be performed on any basic spectrophotometer. Nevertheless, discretionary analysers are still produced which, although excellent in many ways for the performance of conventional spectrophotometric analysis, do not readily perform turbidimetric assays.

The Hitachi 705 has not been recommended for the performance of turbidimetry, however, it is possible to carry out turbidimetric analysis of orosomucoid economically, precisely and rapidly using a selective or batchwise mode of operation. Optimal conditions cannot be used, principally because of timing constraints regarding the addition of the second reagent (antibody). Nevertheless, good precision was obtained using suboptimal conditions, probably reflecting the very accurate timing of which the instrument is capable.

The performance of turbidimetric analysis using the Hitachi 705 renders it a more powerful and versatile instrument in the clinical chemistry laboratory. In addition, this work suggests the probability that other specific protein assays, for example immunoglobulins, can be performed on the Hitachi 705.

### References

1. Price, C. P., Spencer, K. and Whicher, J., *Annals of Clinical Biochemistry*, **20** (1983), 1.
2. Kineiko, R. W., Floering, D. A. and Morrisey, M., *Clinical Chemistry*, **29** (1983), 688.
3. Douville, P. and Forest, J. C., *Clinical Chemistry*, **29** (1983), 692.
4. Buffone, G. P. and Lewis, S. A., *Clinical Chemistry*, **25** (1979), 1009.

### COLLOQUIUM SPECTROSCOPIUM INTERNATIONALE XXIV

*To be held from 15 to 21 September 1985 in Garmisch-Partenkirchen, FR Germany*

CSI XXIV will touch upon all areas of analytical chemistry; an international group of spectroscopists is expected to attend. Programme topics include:

*Basic theory and methods—*
- Atomic emission spectroscopy
- Atomic absorption spectrometry
- Atomic fluorescence spectrometry
- Infra-red and Raman spectroscopy
- Molecular spectroscopy (UV and Vis)
- Laser spectroscopy
- Mass spectroscopy (organic and inorganic)
- Standard reference materials etc.

*Analytical applications for specific problems—*
- Analysis of metals
- Analysis of different industrial products
- Geochemical analysis
- Biological, clinical and pharmaceutical analysis
- Analysis in agriculture and nutrition chemistry
- Environmental analysis.

*The second circular will soon be available from CSI XXIV, Organisationsbüro, Institut für Spektrochemie und angewandte Spektroskopie, Postfach 778, D 4600 Dortmund 1, FR Germany.*

